# Chiral Separation of Cannabichromene, Cannabicyclol, and Their Acidic Analogs on Polysaccharide Chiral Stationary Phases

**DOI:** 10.3390/molecules28031164

**Published:** 2023-01-24

**Authors:** John M. Ferraro, Weston J. Umstead

**Affiliations:** Chiral Technologies, Inc., 1475 Dunwoody Drive, Suite 310, West Chester, PA 19380, USA

**Keywords:** cannabichromene, cannabichromenic acid, cannabicyclol, cannabicyclolic acid, polysaccharide chiral stationary phases, preparative chiral chromatography

## Abstract

Until recently, chirality has not been a major focus in the study of cannabinoids, as most cannabinoids of interest, such as cannabidiol and tetrahydrocannabinol, exist as a single isomer from natural sources. However, this is changing as more cannabinoids are identified, and compounds such as cannabichromene and cannabicyclol are emerging as potential investigatory candidates for varying indications. Because these molecules are chiral, the separation and study of the individual enantiomers’ biological and physiological effects should therefore be of interest. The purpose of this study was to identify analytical separation conditions and then adapt those conditions to preparative separation. This was accomplished with a column-screening approach on Daicel’s immobilized polysaccharide chiral stationary phases using non-traditional mobile phases, which included dichloromethane, ethyl acetate, and methyl tert-butyl ether under high-performance liquid chromatography conditions. CHIRALPAK^®^ IK was found to separate all four compounds well with mobile phases containing hexane-dichloromethane (with or without an acidic additive). From these methods, the separation productivities were calculated to better visualize the separation scalability, which shows that the kilogram-scale separations of each are feasible.

## 1. Introduction

Chirality is a naturally occurring phenomenon that results from the geometric orientation of structural substituents around an atomic center (chiral center) or axis of symmetry. For this to occur, the chiral center must have four different chemical substituents bonded to it, normally in an energetically preferred tetrahedral arrangement. For an axis of symmetry, this means there are two distinct rotational or structural configurations that are hindered in their rotation or movement. The tetrahedral arrangement results in the possibility of two different geometric orientations, referred to as enantiomers. For an axis of symmetry, the two orientations are often referred to as atropisomers. These enantiomers or atropisomers, by virtue of the two distinct configurations, are mirror images, but not superimposable. In simpler terms, imagine your left and right hand!

Within the small molecule pharmaceutical space, chirality has been of particular importance going back to the 1960s and the case of thalidomide [1]. The R enantiomer was a beneficial therapeutic for the treatment of morning sickness; the S enantiomer was a teratogen that caused severe birth defects. One can find countless other examples where chirality has been an important feature of compounds either in a beneficial or deleterious way [2,3,4,5,6,7,8]. Simply put, the structure of molecules and the binding sites they interact with translate to their function, and for this reason, both enantiomers need to be studied to ensure another instance of thalidomide does not occur.

Cannabis contains a complex mixture of compounds, as has been well-documented [9,10,11]. To date, more than 100 unique cannabinoids have been identified [12], many of which exist as only a single isomer. This is for a few potential reasons: (1) the compounds often do not have the possibility of forming chiral centers; this is typically because there are chemical bonds that are not saturated, i.e., the compound contains double bonds; (2) the compounds only have atomic centers with duplicate substituents, for instance, two hydrogens or two carbons; (3) the plant preferentially only synthesizes one of the enantiomers as a requirement for downstream processes. Because most of the compounds of interest (namely cannabidiol (CBD) and tetrahydrocannabinol (THC)) exist naturally as only a single isomer (with some low levels of other isomers having been reported recently [13,14,15]), the literature contains hundreds of examples of separations on classical achiral phases such as silica or octadecyl silane (ODS or C_18_) [16,17,18,19,20].

**Figure 1:**
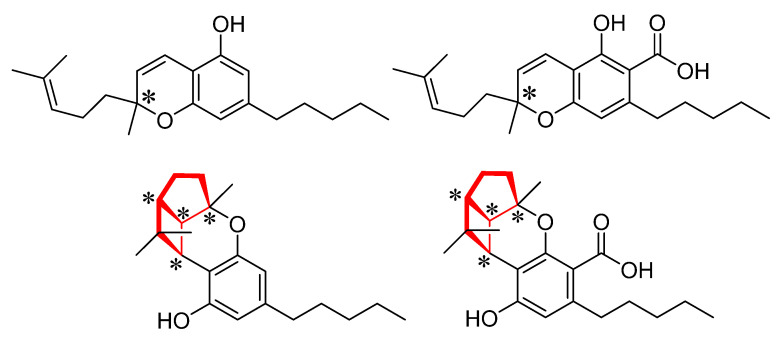
Cannabichromene (**top left**), cannabichromenic acid (**top right**), cannabicyclol (**bottom left**), cannabicyclolic acid (**bottom right**). The starred (*) carbons indicate chiral centers. The red bonds highlight the fused-ring system which results in the chirality of CBL and CBLA.

That being said, there are still some cannabinoids that naturally occur as a pair of enantiomers, such as cannabichromene (CBC) and cannabicyclol (CBL) (Figure 1). The starred carbon centers in Figure 1 represent all chiral centers in the four molecules of this study. They are bonded asymmetrically to four different chemical substituents, therefore making them chiral centers. For cannabicyclol, although it actually has four chiral centers, the enantiomeric pair is formed by the constrained nature of the 4-5-6 membered fused ring system (highlighted in red), which forms a bowl shape that can point cupped forward or backward, creating non-superimposable mirror images. Their acidic analogs, while bearing the addition of a carboxylic acid functional group, retain the same points of chirality as the neutral molecules.

In contrast to the numerous reports on achiral columns, the literature contains considerably fewer examples of chiral separations of cannabinoids, specifically, chiral separations on polysaccharide-based chiral stationary phases (CSPs). There were a handful of studies in the 1990s that looked at the separation of several cannabinoid pairs and their substituted analogs [21,22,23,24,25]. This was followed by a considerable lull until the late 2000s and into the 2010s. More recently, the activity has picked up not only for chiral separations of cannabinoids but also for the achiral separation of cannabinoid mixtures on polysaccharide CSPs [26,27,28,29,30,31,32,33,34,35,36,37,38,39]. Unlike the many non-chiral methods on C18 or silica, chiral phases can achieve a mixed-mode separation of both chiral and non-chiral, making them a powerful tool for complex separations. Beyond the examples in this work, separations such as the analysis of variable THC isomers can be performed using a single-step rather than a multistep method [27,29].

The four compounds in this study have been demonstrated to interact with the CB2 receptor preferentially over the CB1 receptor, which equates to these compounds being minimally psychoactive. Beyond this, the biological and physiological effects have not been well studied, and when they have, it’s been on the racemic mixture, not individual enantiomers. Several studies looking at the potential indications of CBC point to its use for pain relief and inflammation, as well as in stimulating an antinociceptive (pain-blocking) effect in the brain [40,41,42]. Little is presently known with certainty about CBCA and CBL, and much such as CBC, there are indications that CBLA may have anti-inflammatory, antimicrobial, and antioxidative properties [43]. Given that the FDA currently requires a chiral purity assessment and study of the biological effects of single enantiomers in all pharmaceutical applications [44], it is not a stretch to envision a scenario where the same would be required for these compounds.

A previous paper by the author of this work explored the separation of CBC and CBL, with a focus at that time on analytical-only normal-phase high-performance liquid chromatography (HPLC) separations [45]. Separations were reported on several different CSPs using hexane–alcohol mixtures ranging from 5% alcohol by volume to 3% alcohol by volume. Baseline resolution was achieved, but the robustness of such low percentages of alcohol was shown to be problematic, as a critical threshold between 2 and 3% alcohol resulted in severe deterioration of the separations. These mobile phase combinations also present an issue for sample solubility as one tries to adapt these methods for preparative-scale separations. As is shown later in this work, this series of cannabinoids is sparingly soluble in high-alkane-percentage mobile phases. Although chiral separation was not a focus, a related work used similar normal phase solvents for the achiral separation of CBC and several other cannabinoids, exploring the effects the stationary phase polarity had on the reported separations [46].

Therefore, the goal of this work was to revisit these previous separations, improve upon them by exploring non-traditional mobile phase combinations, and adapt said separations for preparative scale to allow for the gram- or kilogram-scale isolation of single enantiomers. As is shared and discussed, this goal was achieved with several immobilized-type polysaccharide CSPs using mainly hexane–dichloromethane mixtures. The use of dichloromethane not only improved the selectivity and resolution from the previously reported methods but also helped improve the solubility of the compounds, which greatly improved the separation productivity.

## 2. Results

### 2.1. Analytical Method Development Screening and Optimization

CBC, CBCA, CBL, and CBLA were screened on extended normal-phase mobile phase mixtures of n-hexane (Hex) with dichloromethane (DCM), ethyl acetate (EtOAc), and methyl tert-butyl ether (MtBE). The reasoning for this is mentioned in Section 1, and is discussed in more detail later in this paper (see Section 3).

**Figure 2:**
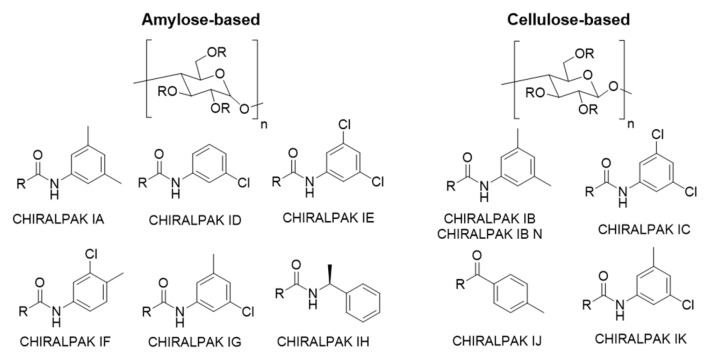
Structures of polysaccharide-based chiral selectors used for method development screening.

The CBC and CBL were prepared as a 1.0 mg/mL solution in ethanol (EtOH) by weighing 1.0 mg of solid (CBL) or thick liquid (CBC) into a 1.5 mL HPLC vial, and diluted with 1.0 mL of EtOH. It should be noted that the best practice normally is to match the sample solvent and mobile phase to ensure that the sample does not crash out on the column when an injection is made. However, for an analytical screening with a low 1.0 mg/mL concentration and only a 5 µL injection, preparation in ethanol is convenient to be able to check many mobile phase combinations without concern for miscibility issues. Ultimately, the sample preparation should be switched to the mobile phase for further optimization and preparative applications, as is discussed in Section 2.2. The samples were then screened on all Daicel’s immobilized polysaccharide-based CSPs, which included CHIRALPAK^®^ IA-3, IB N-3, IC-3, ID-3, IE-3, IF-3, IG-3, IH-3, IJ-3, and IK-3 (structures shown in Figure 2), with the aforementioned solvent mixtures. A screening approach is generally the best place to start for the development of a new chiral method. There is a complex series of intermolecular interactions that can take place on the chiral columns between the chiral analyte and the chiral selector, which makes predicting a column/mobile phase combination difficult and near impossible.

**Figure 3:**
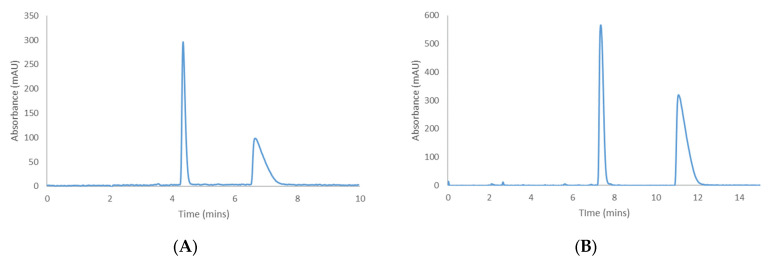
Separation of cannabichromene (**A**) and cannabicyclol (**B**) on CHIRALPAK^®^ IK-3 with Hex−DCM = 80−20 (*v*/*v*).

It was found that Hex-DCM and Hex-MtBE = 80–20 (*v*/*v*) and Hex-EtOAc = 90–10 (*v*/*v*) provided good retention and afforded selectivity for the individual enantiomeric pairs on several columns (Table 1). Figure 3 shows the best separation conditions achieved on CHIRALPAK^®^ IK-3 with Hex-DCM. All other chromatograms for the separations in Table 1 are available in the Supplementary Materials, but IK-3 is specifically highlighted as it was found to be the only CSP that separated all four cannabinoids under their respective screening conditions. The chromatographic performance is shown in Table 2.

**Figure 4:**
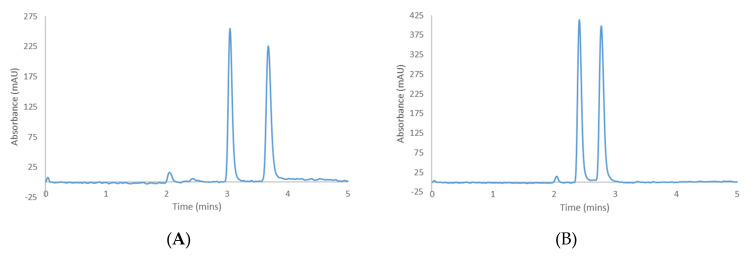
Separation of cannabichromenic acid (**A**) and cannabicyclolic acid (**B**) on CHIRALPAK^®^ IK-3 with Hex−DCM−TFA = 80−20−0.1 (*v*/*v*/*v*).

CBCA and CBLA were similarly prepared as 1.0 mg/mL solutions in EtOH and screened under identical conditions to CBC and CBL, with the addition of 0.1% by volume of trifluoroacetic acid (TFA) to the mobile phase to help improve the peak shape. It was found that Hex-DCM-TFA and Hex-MtBE-TFA = 80–20-0.1 (*v*/*v*/*v*) and Hex-EtOAc-TFA = 90–10-0.1 (*v*/*v*/*v*) provided good retention and afforded selectivity for the individual enantiomeric pairs on several columns (Table 3). Figure 4 shows the best separation conditions achieved on CHIRALPAK^®^ IK-3 with Hex-DCM-TFA. All other chromatograms for the separations in Table 2 are also available in the Supplementary Materials, but IK-3 is again specifically highlighted as it was found to be the only CSP that separated all four cannabinoids under their respective screening conditions.

The chromatographic performance is shown in Table 4, where k is the retention factor, α is the selectivity, and R_s_ is the resolution.

### 2.2. Preparative Method Development Optimization and Productivity Determination

CHIRALPAK^®^ IK was the CSP chosen for further preparative optimization, as it was the only CSP found to separate all four compounds with a similar mobile phase. While the other columns certainly provided similar or perhaps better selectivity and resolution, or offered slightly better solubility, there are practical merits to unifying column and method conditions whenever possible. It should be additionally noted that all conditions in Table 1 and Table 2 could be further optimized in a similar manner as described below to provide for preparative separation conditions on other columns. Based on the findings for the optimization of IK separations, the general trend for the cannabinoids in this study would be more organic solvent results in faster elution, and vice versa.

The method development screening was performed on columns with a 3 µm particle size. A 5 µm particle size is the smallest recommended particle size for preparative applications, so the first step of optimization was to rerun the screening conditions on CHIRALPAK IK, the 5 µm equivalent of IK-3. The column length was increased from a length of 150 mm length to 250 mm. The 150 mm length columns can provide a faster initial screening, but a 250 mm length is often more desirable for preparative applications given the increased resolution. No changes were made to the flow rate in moving to the longer column length. In theory, one could increase the flow rate by a factor of 1.67× (the ratio of the new column length to the old column length). This would result in similar elution times and similar resolutions. However, one of the purposes of moving to a longer column is to increase the resolution, so 1.0 mL/min was maintained throughout the screening and optimization.

**Figure 5:**
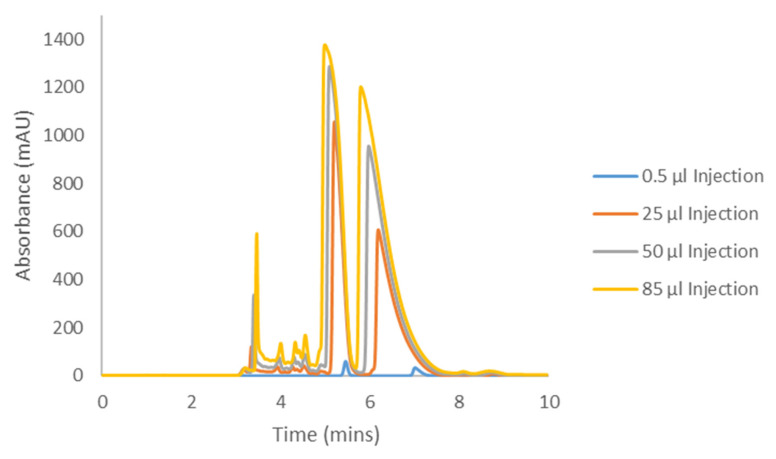
Preparative loading of CBC with a sample concentration of 25.7 mg/ml on CHIRALPAK^®^ IK with Hex−DCM = 60−40.

**Figure 6:**
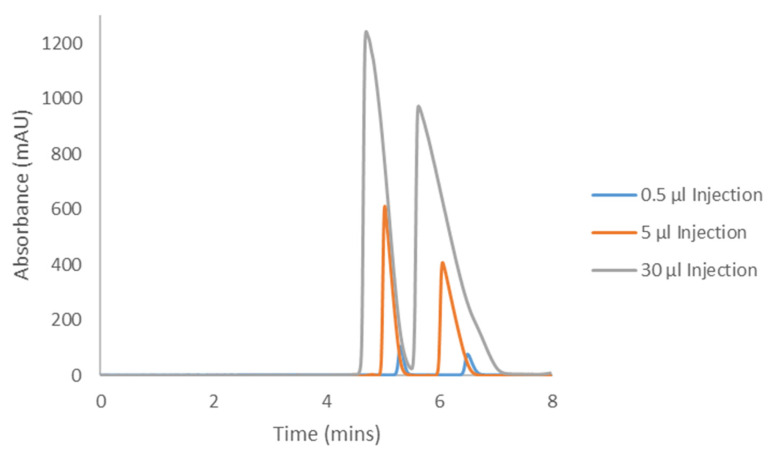
Preparative loading of CBCA with a sample concentration of 25.0 mg/mL on CHIRALPAK^®^ IK with Hex−DCM−TFA = 80−20−0.1.

**Figure 7:**
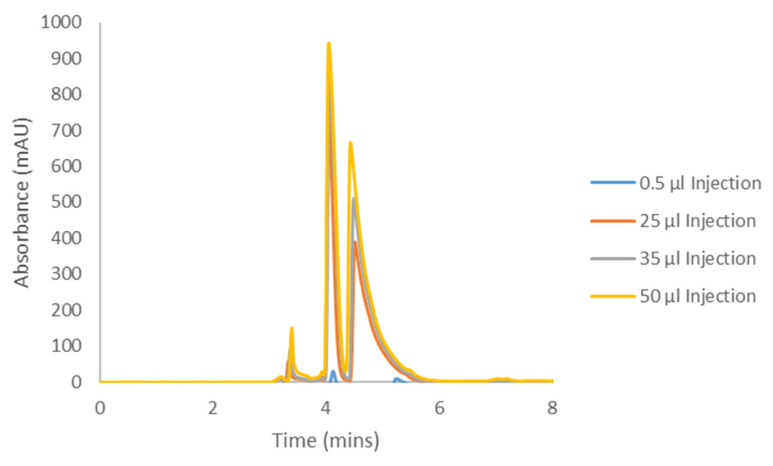
Preparative loading of CBL with a sample concentration of 19.0 mg/mL on CHIRALPAK^®^ IK with Hex−DCM = 60−40.

**Figure 8:**
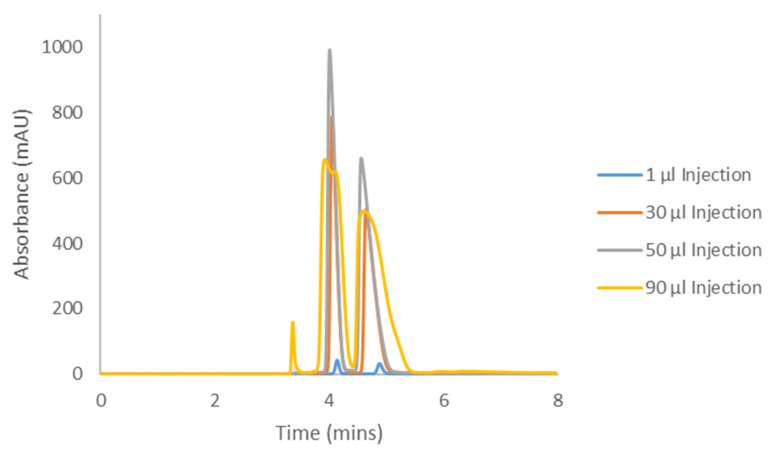
Preparative loading of CBLA with a sample concentration of 3.5 mg/mL on CHIRALPAK^®^ IK with Hex−DCM−TFA = 80−20−0.1.

The Hex−DCM(−TFA) methods from Table 1 and Table 2 were selected as appropriate mobile phase conditions as they afforded separation on IK for all cannabinoids studied. The methods were repeated on the longer columns as mentioned above to generate representative “analytical-like” chromatograms for the adjusted conditions, before increasing the injection volumes to determine the compound loading. These analytical injections are shown in Figure 5, Figure 6, Figure 7 and Figure 8 as the blue 0.5 and 1.0 µL traces. During this initial optimization, it was found that the retentions of CBC and CBL became unnecessarily long when moving to the 250 mm length column. The addition of DCM, the more polar mobile phase component, eluted CBC and CBL in a more reasonable amount of time, while maintaining good selectivity and resolution. For this reason, when comparing the elution times and elution orders between Table 3 and Table 4, there are differences arising from this additional optimization. Because of the lower selectivity for the CBCA and CBLA methods, the original screening mobile phase of Hex−DCM = 80−20 was maintained.

The performance for each compound on these methods, including the retention factor (k), selectivity (α), and resolution (R_s_), are shown in Table 5. Note that the retention times are not corrected for the system dead volume as the same instrument was used for the entire study (screening and optimization). One would expect some variation when reproducing these separations on a different instrument, depending on factors such as the system’s tubing lengths and tubing inner diameters.

Samples of the four compounds were prepared in the corresponding Hex−DCM mobile phase at varying concentrations based on the compound solubility. This was determined by weighing a set amount into a 1.5 mL HPLC vial and adding mobile phase (with occasional vortexing) until the entire sample was dissolved. CBC was soluble in mobile phase (MP) at greater than 25 mg/mL (it is an oil, so its solubility was likely much higher than reported here), CBCA was soluble at 140.6 mg/mL, CBL was soluble at 24.8 mg/mL, and CBLA was soluble at 4.26 mg/mL. The compound solubilities often resulted in only a few hundred microliters of solution, so in order to have enough of the samples for subsequent loading studies, these maximum concentration samples were further diluted to yield 1 mL of the final solution. The final concentrations are reported in each figure caption (Figure 5, Figure 6, Figure 7 and Figure 8).

These solubilities are comparably better than those in the previously utilized hexane/alcohol mobile phases, particularly for CBCA, CBL, and CBLA (Table 6). For real-world extracts, where the actual concentrations of these cannabinoids are relatively low, the solubility is likely not a problem. However, for synthetically produced materials, such as those used in this work, where the theoretical goal was to isolate as much pure enantiomer as possible in the shortest amount of time, this solubility improvement is important.

Each cannabinoid sample was then injected into the HPLC system using the optimized preparative method conditions in increasing injection volumes until the back of peak 1 started to coelute with the front of peak 2, otherwise known as the “touching-band” approach (Figure 5, Figure 6, Figure 7 and Figure 8). The increasing volume of this series of injections inevitably led to the saturation of the detector, so the wavelength used for analytical detection needed to be adjusted to avoid this. Saturating a detector can falsely show column overloading before it actually occurs, thus resulting in less productivity. To determine what wavelength to use, each compound was injected into the HPLC system bypassing the column (also referred to as a flow injection) and the UV profile was evaluated. All compounds were found to be absorbed well at 230 nm, although 230 nm is not the global UV maximum for each compound. All compounds also showed a local UV maximum of around 250–270 nm, which decayed to no UV activity beyond that point (variable for each compound). Based on these individual flow injections, a UV detection wavelength of 330 nm was chosen for CBC, 370 nm for CBCA, 290 nm for CBL, and 350 nm for CBLA. These higher wavelengths ensured the sample could be seen, but the signal was not strong enough to saturate the detector and falsely appeared as overloaded.

Using the sample concentration, the injection volume, and the cycle time (the time for peak 1 to start eluting and peak 2 to finish eluting), the productivity in milligrams per hour was determined using Equation 1. Indeed, there was some dead time before the elution of peak 1 from the first injection; however, the assumption for this calculation was that the injections are stacked. Stacked injections will eliminate this dead time in the subsequent injections, limiting the impact of this time on the productivity overall.

(1) $$Productivity\;\left(\frac{mg}{hr}\right)=\frac{Injection\;Volume\;\left(ml\right)\times Concentration\;\left(\frac{mg}{ml}\right)}{Cycle\;Time\;\left(hrs\right)}$$

On an analytical column with a length of 250 mm (mm) length (L) and inner diameter (i.d.) of 4.6 mm, CBC was determined to have a productivity of 43.69 mg/hour, CBCA had a productivity of 15.52 mg/hour, CBL had a productivity of 28.5 mg/hour, and CBLA had a productivity of 9.95 mg/hour. Additional details of scaling to larger inner diameter preparative columns are provided in Section 3.

Lastly, the fractions of peak 1 and peak 2 for the representative compounds were collected from the loading experiments and assessed for stability. Aliquots were held at ambient temperatures of 30 °C and 50 °C and either uncapped (allowed to evaporate freely) and capped (in solution for the duration of the study) for 18 h. After 18 h, the samples were reinjected, using the analytical method to visualize any degradation or racemization. For all four compounds, no issues were observed after the 18 h hold, indicating that preparative scaling up should not be problematic.

## 3. Discussion

As briefly mentioned in Section 2, previous work has been performed to assess the chiral resolutions of CBC and CBL under analytical-only separation conditions [45]. Baseline separations were achieved for both CBC and CBL using CHIRALPAK^®^ IB N-3, which is an immobilized cellulose tris (3,5-dimethylphenylcarbamate) chiral selector, using traditional normal-phase alkane/alcohol mixtures. While alkane/alcohol mixtures can be favorable to use for a number of reasons, the separations of CBC and CBL were found to require a very low percentage of alcohol (3–5% by volume) to achieve sufficient retention for selectivity. This can be problematic for method robustness as was demonstrated in several of those examples [45]. In most cases, there was shown to be a critical alcohol percentage required for good separation and peak shape (typically 3–4% by volume); anything less and the peak shape and separation were lost entirely.

To separate from and improve upon that previous work, the use of non-traditional or “extended range” mobile phases was explored on Daicel’s immobilized i-series of columns. This was for two reasons: (1) The extended range solvents, such as DCM, EtOAc, and MtBE, can provide new and improved separations because of changes to the on-column intermolecular interactions [47,48], and (2) these solvents often provide better solubility compared to traditional normal-phase alkane/alcohol combinations. The second reason is of particular importance when considering preparative separation conditions, as solubility is one of the primary factors that can positively or negatively affect the method’s productivity. While in theory, one should be able to inject more of a dilute sample into a column before the column becomes overloaded, certain limitations, such as the sample loop size, can limit the maximum productivity for very dilute samples.

Before thoroughly discussing the results of the productivity studies, it is critical to note that productivity scales are proportional to the ratio of the column’s inner diameters squared, as given in Equation 2. The equation as written requires both columns to have the same length, otherwise, an additional ratio to account for the column lengths would need to be included. This scaling factor also applies to the injection volumes and flow rates for the separation.

(2) $$Scaling\;Factor=\frac{{\left(Column\;1\;inner\;diameter\right)}^{2}}{{\left(Column\;2\;inner\;diameter\right)}^{2}}$$

This can make predicting the productivity and adapting the analytical methods to the preparative size column dimensions fairly straightforward. This equation was used for each compound in this work, and the productivities shared in Section 2 are presented below and scaled to several common preparative column sizes for easy visualization. Also presented is the productivity in kilograms (kg) of racemate per kg of CSP per day (kg racemate/kg CSP/day) (Equation 3). This can be determined by knowing the amount of CSP contained in a given column dimension, for example, a 4.6 × 250 mm analytical column, as used in this work, contains 2.5 g of CSP.

(3) $$Productivity\;(kg\;of\;racemate\;per\;kg\;of\;CSP\;per\;day=Productivity\;\left(\frac{kg}{hr}\right)\times Mass\;of\;CSP\;\left(kg\right)\times Hours\;in\;a\;Work\;Day$$

For scaling beyond a prepacked preparative column, this value is useful to approximate the productivity on any size system or column so long as the CSP requirement for that system (in grams or kg) is known. This value is often used for appropriately sizing a simulated moving bed (SMB) chromatography system for example.

One final note before discussing the individual preparative results—method validation was not a focus of this work, and therefore, no formal presentation of that process is presented here. However, over the course of the analytical and preparative development and optimization, several column lots, specifically, several CSP lots, were used. Good reproducibility and repeatability were found across these lots, so at least anecdotally these methods all appear to be robust.

### 3.1. Cannabichromene Preparative Chiral Resolution

CBC had the best productivity by far of all four compounds used in this study. This was due in part to the very good solubility, but also a desirable loading. That is, peak 1 and peak 2 did not start coeluting until a much higher on-column concentration was achieved. Chiral molecules can “load” in a variety of different ways on a chiral column depending on the compound-specific isotherm [49,50]. Ideally, as more material is loaded onto the column, a symmetrical, Gaussian peak shape from the analytical chromatography will be retained, and the peaks will simply grow larger. This is referred to as a linear isotherm. Quite often, however, as we move into a range of higher on-column concentrations that maximize productivity, the peak shape begins to tail, which would indicate a Langmuir isotherm. The opposite can also happen, where peaks begin to front, which is referred to as an anti-Langmuir isotherm. The peak shape can also begin to deteriorate in a combination of ways, often referred to as a Quadratic isotherm. These isotherms can be determined experimentally and plotted as a function of the retention factor versus the on-column concentration, but they can also be readily visualized via the overloading studies presented below.

As more CBC was injected into the column, we observed Langmuir behavior, that is the peaks began to tail. The loading injections for the CBC separation are shown in Figure 5. The productivity, as determined on the 4.6 × 250 mm analytical column, was 43.69 mg/hr. This was the expected productivity of the input racemate. One could also estimate the excepted yield or recovery of the separated enantiomers based on this. A total of 50% of the productivity was 21.84 mg/hr, which is the productivity of a single enantiomer; yields for most HPLC systems are in the 85–95% range, so one could expect between 18.56 mg and 20.74 mg/hr of separated enantiomer peaks 1 and 2 on a 4.6 × 250 mm column for this method. When using Equation 2, we found that the scaling factors for common preparative column sizes were ~20 (for 2 cm i.d.), ~40 (for 3 cm i.d.), and ~120 (for 5 cm i.d.). The scaled productivities are presented in Table 7 and show that gram-scale isolation was feasible on a daily basis. Note that the calculation for the kg racemate/kg of CSP/day assumed a 16 h day.

### 3.2. Cannabichromenic Acid Preparative Chiral Resolution

A general finding from this study was that the solubility of the acidic cannabinoids was much lower than their neutral analogs in similar mobile phases. The solubility of CBC (given that it is an oil) is theoretically infinite, whereas the solubility of CBCA was determined to be 140.6 mg/mL in mobile phase. This is still an excellent solubility as far as preparative method development is concerned; however, a stark contrast exists. The productivity for CBCA was roughly 65% less than that for CBC, and calculated to be 15.52 mg/hr on the 4.6 × 250 mm analytical column (Table 8). For individual enantiomer recovery, this is between 6.59 mg and 7.37 mg per hour on a 4.6 × 250 mm analytical column.

Two factors contributed to this lower productivity: (1) a decrease in the method selectivity and resolution compared to CBC, and (2) the “speed” at which peak 1 and peak 2 begin to coelute. CBCA exhibited Langmuir behavior similar to CBC, but the decrease in selectivity resulted in the front of peak 2 coeluting more quickly with peak 1 (Figure 6). In spite of this decrease, a large enough preparative system could still afford gram quantities of separated material on a daily basis (nearly 10 g of racemate throughput on a 3 cm i.d. column, or up to 4.7 g of pure enantiomer (assuming a 16 h work day and 95% recovery)).

### 3.3. Cannabicyclol Preparative Chiral Resolution

Returning to a neutral cannabinoid, CBL had modest solubility in the mobile phase, but still much less than CBC. As noted in Section 2, this 24.25 mg/mL solubility is still considerably better than the < 5 mg/mL solubility in the previously reported hexane/alcohol mixtures [45]. CBL exhibited Langmuir behavior as it was overloaded, and while productivity was not as high as CBC, it was better than CBCA (Table 9). This was primarily due to the improvements in the loading coming from a higher resolution (compared to CBCA), and the slower rate at which the forward movement of peak 2 occurred (Figure 8). The adjusted recovery (85–95% yield) for individual enantiomers would be 11.68 mg to 13.53 mg/hr on a 4.6 × 250 mm analytical column.

### 3.4. Cannabicyclolic Acid Preparative Chiral Resolution

The last compound in the series, CBLA, exhibited the worst solubility of the four compounds studied, but again, it was still better at 4.2 mg/mL in Hex-DCM-TFA than <2 mg/mL in Hex-EtOH. CBLA also exhibited Langmuir behavior when overloaded—one might notice, however, the peak shape deterioration as well. The peaks began to split as the column was overloaded, which is not unusual (but also not predictable). The productivity was severely limited at 9.95 mg/hr on a 4.6 × 250 mm analytical column (Table 10). The adjusted recoveries (85–95% yield) for single enantiomers would be between 4.23 mg and 4.73 mg/hr. Because of the low solubility, the method for CBLA does allow for a much higher injection volume compared to some other methods (90 µL, Figure 8); however, CBLA still exhibited the lowest productivity of the four compounds in this study. In spite of this, similar to CBCA, a large enough preparative system could still provide gram quantities of material daily, which is encouraging.

## 4. Materials and Methods

KinetoChem LLC in Georgetown, TX kindly provided the four racemic synthetically prepared cannabinoids. These included cannabichromene (CBC), cannabichromenic acid (CBCA), cannabicyclol (CBL), and cannabicyclolic acid (CBLA). The cannabinoids were prepared as 1.0 milligram per milliliter (mg/mL) samples in ethanol (EtOH), and screened on Daicel’s immobilized chiral stationary phases (CSPs), which included CHIRALPAK^®^ IA-3 (amylose tris (3,5-dimethylphenylcarbamate)), IB N-3 (cellulose tris (3,5-dimethylphenylcarbamate)), IC-3 (cellulose tris (3,5-dichlorophenylcarbamate)), ID-3 (amylose tris (3-chlorophenylcarbamate)), IE-3 (amylose tris (3,5-dichlorophenylcarbamate)), IF-3 (amylose tris (3-chloro-4-methylphenylcarbamate)), IG-3 (amylose tris (3-chloro-5-methylphenylcarbamate)), IH-3 (amylose tris (S)-α-methylbenzylcarbamate), IJ-3 (cellulose tris 4-methylbenzoate)), and IK-3 (cellulose tris (3-chloro-5-methylphenylcarbamate)). All columns for screening had a 3 µm particle size with dimensions of 150 mm L × 4.6 mm i.d. For the preparative optimization, all columns had a 5 µm particle size with dimensions of 250 mm L × 4.6 mm i.d.

All screening and method optimization was performed on an Agilent 1200 HPLC (Agilent Technologies, Delaware, United States) configured with low-pressure mixing, a quaternary mobile phase delivery system, a vacuum degasser, an autosampler, and a photodiode array UV detector. The instrument was controlled by an Agilent ChemStation Version RevB.04.03 [16].

All solvents were of HPLC grade or higher and purchased from the Scientific Equipment Company (Aston, PA, USA). Specifically, the hexanes used contained 95% n-hexane, and the ethanol was reagent alcohol (90% ethanol with 5% methanol and 5% isopropanol *v*/*v*/*v*). Trifluoroacetic acid (TFA) was purchased from Sigma Aldrich (Allentown, PA) and used as is. The other experimental parameters, such as the temperature, flow rate, and injection volume, varied across the different analytical and preparative methods, and are indicated in Table 1 and Table 2.

## 5. Conclusions

Historically, chirality has not been a major focus for Cannabis research. Many of the key compounds, until recently, have occurred naturally as a single isomer, so the chiral analysis and preparative isolation have not occurred to the same degree as, for example, the isolation and removal of THC from hemp extract. However, with the identification of more compounds, such as CBC and CBL, and the discovery of other THC isomers, such as Δ^6^ and Δ^10^ THC, the need for chiral analysis and preparative isolation should also continue to grow. Particularly, as we move towards potential legalization, it would not be a stretch to imagine a governing body such as the FDA requiring a biological assessment of the pure enantiomers (such as they do for pharmaceuticals). This paper provides separation methods for CBC, CBCA, CBL, and CBLA that are capable of providing analytical enantiomeric analysis and preparative quantities sufficient for studying their biological and physiological effects.

The methods presented in this work improve upon previous methods using traditional normal-phase alkane/alcohol mixtures by increasing the solubility in the mobile phases and improving the selectivity. As many cannabinoids are structurally related and potentially suffer from the same issues as these four compounds (low solubility in the traditional normal phase for example), this work provides non-traditional mobile phase combinations that can be helpful in overcoming those limitations. It should be noted that only synthetically generated standards were used for this work—a real-world extract would likely prove a bit challenging given the sample complexity and lower concentrations. With the specificity at which synthetically generated cannabinoids can be manufactured, this is likely not a prohibitive issue, where samples are less complex and working concentrations for preparative separations are much higher than for extracts.

Daicel Corporate Disclaimer: As a responsible provider of quality products and services, Daicel Chiral Technologies provides analytical techniques, which may be of use to a broad range of customers and applications. It does not, however, support or promote the use of its products or services in connection with any contraband activities or products related to Cannabis; this includes, but is not limited to, illegal or illicit drug manufacturing, testing, or consumption.

## Figures and Tables

**Table 1:** Summary of analytical screening results yielding separations of CBC and CBL.

	Hex-DCM	Hex-MtBE	Hex-EtOAc
Column and Dimensions	CHIRALPAK^®^ IC-3, IG-3, and IK-3 (150 mm L × 4.6 mm i.d., 3 µm)	CHIRALPAK^®^ IB N-3, IG-3, and IK-3 (150 mm L × 4.6 mm i.d., 3 µm)	CHIRALPAK^®^ IB N-3, and IK-3 (150 mm L × 4.6 mm i.d., 3 µm)
Mobile Phase Ratio	80–20 (*v*/*v*)	80–20 (*v*/*v*)	90–10 (*v*/*v*)
Flow Rate	1.0 mL/min		
Temperature	25 °C (controlled)		
Detection	230 nm UV (for DCM and MtBE) 280 nm UV (for EtOAc)		
Sample	1.0 mg/mL in EtOH		
Injection Volume	5 µl		

**Table 2:** Chromatographic performance of analytical cannabinoid separations on IK with Hex-DCM (-TFA) = 80-20 (-0.1) mobile phases.

	RT_1_	RT_2_	*k*_1_^a^	*k*_2_^a^	α	R_s_
CBC	7.35 min	11.08 min	3.26	5.42	1.66	4.24 ^b^
CBCA	3.05 min	3.68 min	0.77	1.13	1.47	4.19 ^c^
CBL	4.34 min	6.65 min	1.51	2.85	1.88	3.40 ^d^
CBLA	2.41 min	2.77 min	0.40	0.60	1.53	2.64 ^e^

RT = retention time. ^a^ Column void time (t_0_) was determined to be 1.727 min using 1,3,5-tri-tert-butylbenzene (TTBB). ^b,c,d,e^ Plate counts determined to be 2558, 9742, 1535, and 6613 plates.

**Table 3:** Summary of analytical screening results yielding separations for CBCA and CBLA.

	Hex-DCM-TFA	Hex-MtBE-TFA	Hex-EtOAc-TFA
Column and Dimensions	CHIRALPAK^®^ IC-3 and IK-3 (150 mm L × 4.6 mm i.d., 3 µm)	CHIRALPAK^®^ IK-3 (150 mm L × 4.6 mm i.d., 3 µm)	CHIRALPAK^®^ IK-3 (150 mm L × 4.6 mm i.d., 3 µm)
Mobile Phase Ratio	80–20-0.1 (*v*/*v*/*v*)	80–20-0.1 (*v*/*v*/*v*)	90–10-0.1 (*v*/*v*/*v*)
Flow Rate	1.0 mL/min		
Temperature	25 °C (controlled)		
Detection	230 nm UV (for DCM and MtBE) 280 nm UV (for EtOAc)		
Sample	1.0 mg/mL in EtOH		
Injection Volume	5 µl		

**Table 4:** Chromatographic performance of analytical cannabinoid separations on IK with Hex−DCM(−TFA) = 80−20(−0.1) mobile phases.

	RT_1_	RT_2_	*k*_1_^a^	*k*_2_^a^	α	R_s_
CBC	7.35 min	11.08 min	3.26	5.42	1.66	4.24 ^b^
CBCA	3.05 min	3.68 min	0.77	1.13	1.47	4.19 ^c^
CBL	4.34 min	6.65 min	1.51	2.85	1.88	3.40 ^d^
CBLA	2.41 min	2.77 min	0.40	0.60	1.53	2.64 ^e^

RT = retention time. ^a^ Column void time (t_0_) was determined to be 1.727 min using 1,3,5-tri-tert-butylbenzene (TTBB). ^b,c,d,e^ Plate counts determined to be 2558, 9742, 1535, and 6613 plates.

**Table 5:** Chromatographic performance of preparative cannabinoid separations on IK with Hex−DCM(−TFA) = 60−40 or 80−20(−0.1) mobile phases.

	RT_1_	RT_2_	*k*_1_^a^	*k*_2_^a^	α	R_s_
CBC	5.45 min	7.01 min	1.00	1.58	1.58	5.42 ^b^
CBCA	5.31 min	6.50 min	0.95	1.39	1.46	5.05 ^c^
CBL	4.13 min	5.24 min	0.51	0.93	1.82	5.92 ^d^
CBLA	4.14 min	4.88 min	0.52	0.79	1.52	6.06 ^e^

RT = retention time. ^a^ Column void time (t_0_) was determined to be 2.741 min using 1,3,5-tri-tert-butylbenzene (TTBB). ^b,c,d,e^ Plate counts determined to be 9288, 11,660, 6621, and 13,800 plates.

**Table 6:** Solubility comparison of cannabinoids in traditional and non-traditional normal-phase mobile phases.

	Hex−EtOH = 95−5 (*v*/*v*)	Hex−DCM = 80−20 (*v*/*v*)	Hex−EtOAc = 90−10 (*v*/*v*)	Hex−MtBE = 80−20 (*v*/*v*)
CBC	*n.d.*	*n.d.*	*n.d.*	*n.d.*
CBCA	28.2 mg/mL	140.6 mg/mL	29.1 mg/mL	87.3 mg/mL
CBL	<5 mg/mL	24.8 mg/mL	18.7 mg/mL	19.3 mg/mL
CBLA	<2 mg/mL	4.3 mg/mL	7.2 mg/mL	11.4 mg/mL

n.d. = Not determined as the compound was an oil.

**Table 7:** Productivity of CBC separation on various column dimensions.

	4.6 × 250 mm	21 × 250 mm	30 × 250 mm	50 × 250 mm	kg Racemate/kg of CSP/Day
CBC Productivity	43.69 mg/hr	873.8 mg/hr	1.75 g/hr	5.24 g/hr	0.279 kg/kg CSP/day

**Table 8:** Productivity of CBCA separation on various column dimensions.

	4.6 × 250 mm	21 × 250 mm	30 × 250 mm	50 × 250 mm	kg Racemate/kg of CSP/Day
CBCA Productivity	15.52 mg/hr	310.4 mg/hr	620.8 mg/hr	1.86 g/hr	0.099 kg/kg CSP/day

**Table 9:** Productivity of CBL separation on various column dimensions.

	4.6 × 250 mm	21 × 250 mm	30 × 250 mm	50 × 250 mm	kg Racemate/kg of CSP/Day
CBL Productivity	28.5 mg/hr	570 mg/hr	1.14 g/hr	3.42 g/hr	0.182 kg/kg CSP/day

**Table 10:** Productivity of CBLA separation on various column dimensions.

	4.6 × 250 mm	21 × 250 mm	30 × 250 mm	50 × 250 mm	kg Racemate/kg of CSP/Day
CBLA Productivity	9.95 mg/hr	199 mg/hr	398 mg/hr	1.19 g/hr	0.064 kg/kg CSP/day

## Data Availability

The data are contained within the article. The data presented in this study are available in *Analytical and Preparative Chiral Separation of Cannabichromene, Cannabicyclol, and their Acidic Analogs on Polysaccharide Chiral Stationary Phases*.

## Supplementary Materials

The following supporting information can be downloaded at: https://www.mdpi.com/article/10.3390/molecules28031164/s1, Figure S1: Separation of CBC on CHIRALPAK^®^ IB N-3 with Hex-MtBE = 80-20 (*v*/*v*); Figure S2: Separation of CBC on CHIRALPAK^®^ IB N-3 with Hex-EtOAc = 90-10 (*v*/*v*); Figure S3: Separation of CBC on CHIRALPAK^®^ IC-3 with Hex-MtBE = 80-20 (*v*/*v*); Figure S4: Separation of CBC on CHIRALPAK^®^ IG-3 with Hex-DCM = 80-20 (*v*/*v*); Figure S5: Separation of CBC on CHIRALPAK^®^ IG-3 with Hex-MtBE = 80-20 (*v*/*v*); Figure S6: Separation of CBC on CHIRALPAK^®^ IK-3 with Hex-EtOAc = 90-10 (*v*/*v*); Figure S7: Separation of CBC on CHIRALPAK^®^ IK-3 with Hex-MtBE = 80-20 (*v*/*v*); Figure S8: Separation of CBC on CHIRALPAK^®^ IC-3 with Hex-DCM = 80-20 (*v*/*v*); Figure S9: Separation of CBCA on CHIRALPAK^®^ IC-3 with Hex-DCM-TFA = 80-20-0.1 (*v*/*v*/*v*); Figure S10: Separation of CBCA on CHIRALPAK^®^ IK-3 with Hex-MtBE-TFA = 80-20-0.1 (*v*/*v*/*v*); Figure S11: Separation of CBCA on CHIRALPAK^®^ IK-3 with Hex-EtOAc-TFA = 90-10-0.1 (*v*/*v*/*v*); Figure S12: Separation of CBL on CHIRALPAK^®^ IBN-3 with Hex-EtOAc = 90-10 (*v*/*v*); Figure S13: Separation of CBL on CHIRALPAK^®^ IBN-3 with Hex-MtBE = 80-20 (*v*/*v*); Figure S14: Separation of CBL on CHIRALPAK^®^ IC-3 with Hex-DCM = 80-20 (*v*/*v*); Figure S15: Separation of CBL on CHIRALPAK^®^ IG-3 with Hex-DCM = 80-20 (*v*/*v*); Figure S16: Separation of CBL on CHIRALPAK^®^ IG-3 with Hex-EtOAc = 90-10 (*v*/*v*); Figure S17: Separation of CBL on CHIRALPAK^®^ IG-3 with Hex-MtBE = 80-20 (*v*/*v*); Figure S18: Separation of CBLA on CHIRALPAK^®^ IC-3 with Hex-DCM-TFA = 80-20-0.1 (*v*/*v*/*v*); Figure S19: Separation of CBLA on CHIRALPAK^®^ IG-3 with Hex-EtOAc-TFA = 90-10-0.1 (*v*/*v*/*v*); Figure S20: Separation of CBLA on CHIRALPAK^®^ IK-3 with Hex-MtBE-TFA = 80-20-0.1 (*v*/*v*/*v*).

## Sample Availability

Samples of the compounds are not available or not from the authors.
