# Supplementary material for: Chiral Separation of Cannabichromene, Cannabicyclol, and Their Acidic Analogs on Polysaccharide Chiral Stationary Phases
Source: Molecules. 2023 Jan 24;28(3):1164. doi: 10.3390/molecules28031164 (PMC9921479; doi:10.3390/molecules28031164)
Supplement: Supplementary file 1 [file molecules-28-01164-s001.zip › molecules-2120985-supplementary.pdf]

Supplementary

Chiral Separation of Cannabichromene, Cannabicyclol, and their Acidic Analogs on Polysaccharide Chiral Stationary Phases

John M. Ferraro [1], Dr. Weston J. Umstead [1,*]

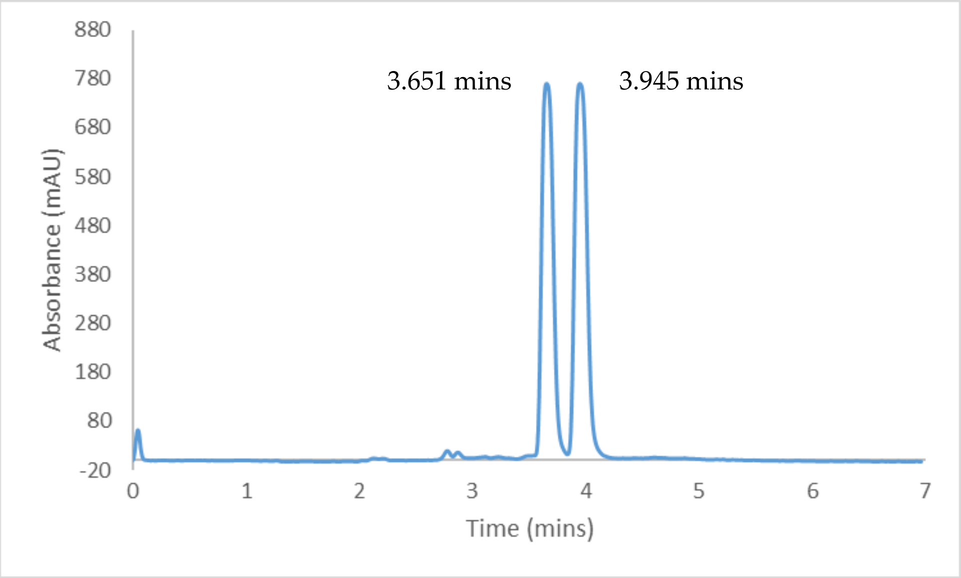

Figure S1: Separation of CBC on CHIRALPAK® IB N-3 with Hex-MtBE = 80-20 (v/v)

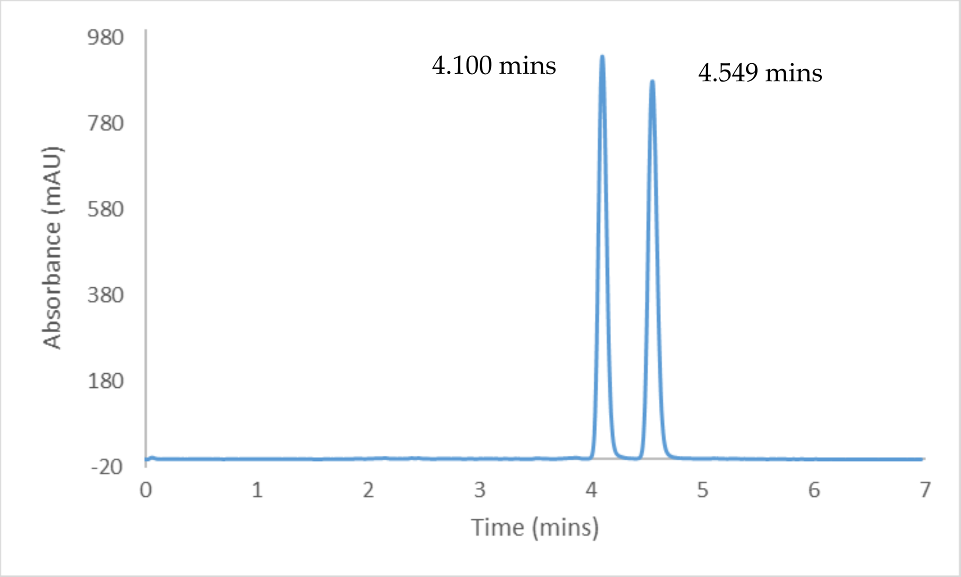

Figure S2: Separation of CBC on CHIRALPAK® IB N-3 with Hex-EtOAc = 90-10 (v/v)

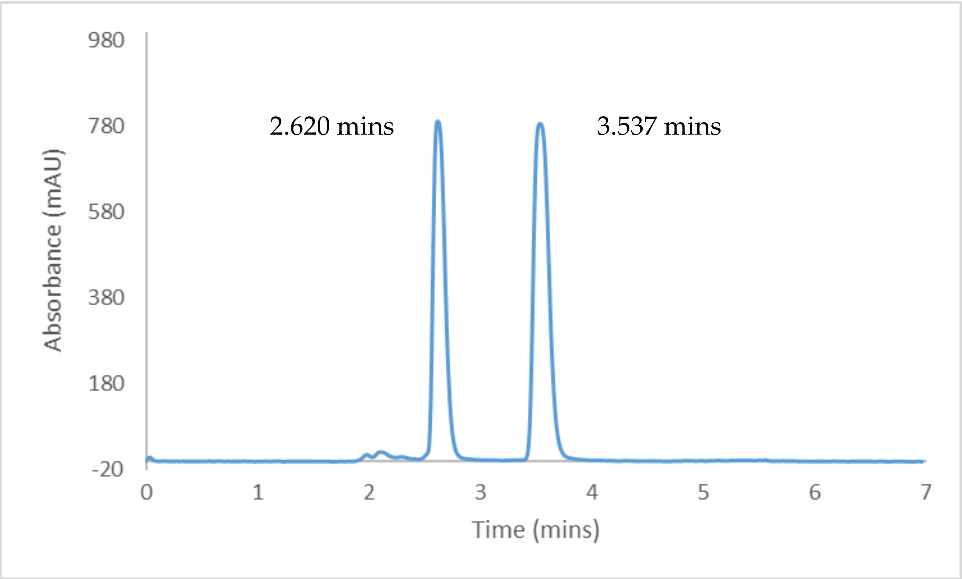

Figure S3: Separation of CBC on CHIRALPAK® IC-3 with Hex-MtBE = 80-20 (v/v)

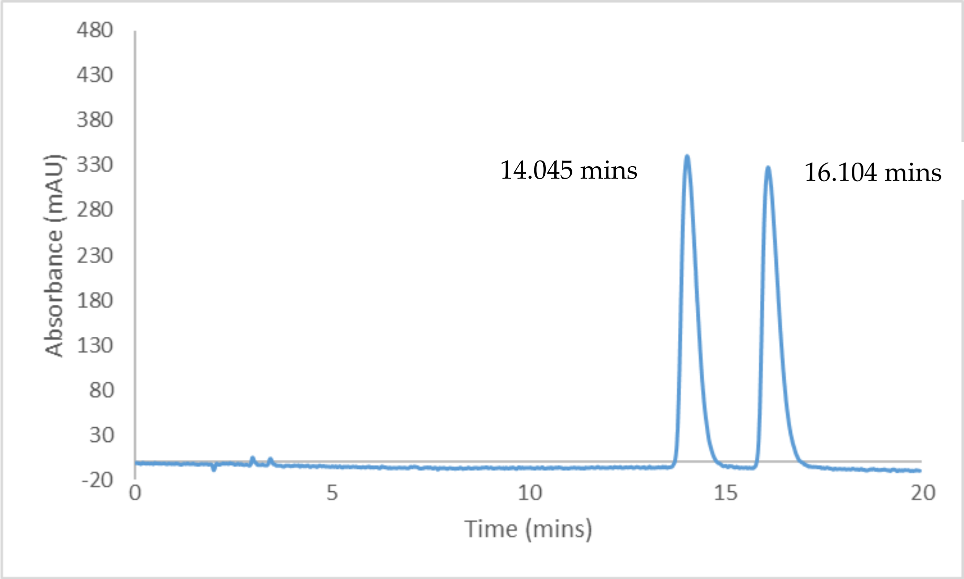

Figure S4: Separation of CBC on CHIRALPAK® IG-3 with Hex-DCM = 80-20 (v/v)

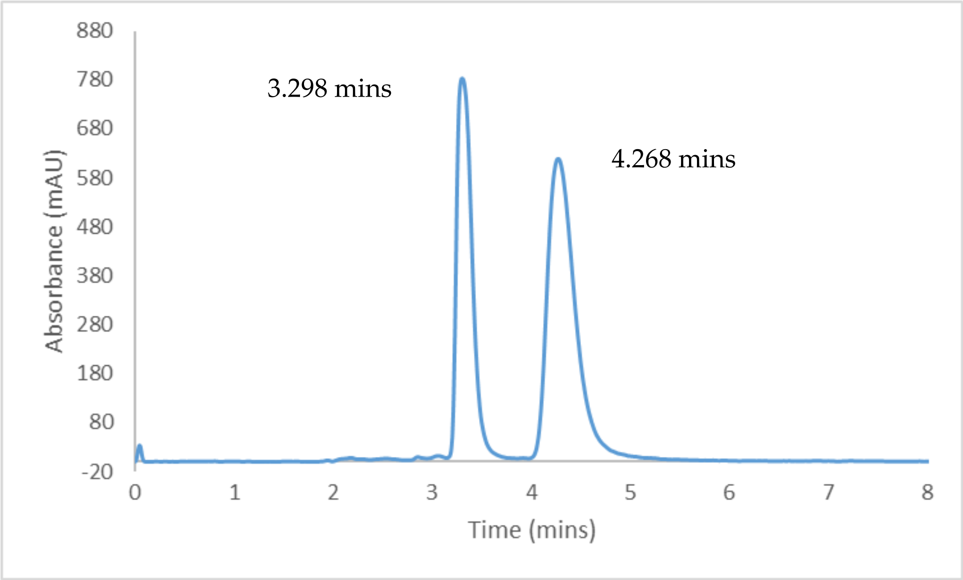

Figure S5: Separation of CBC on CHIRALPAK® IG-3 with Hex-MtBE = 80-20 (v/v)

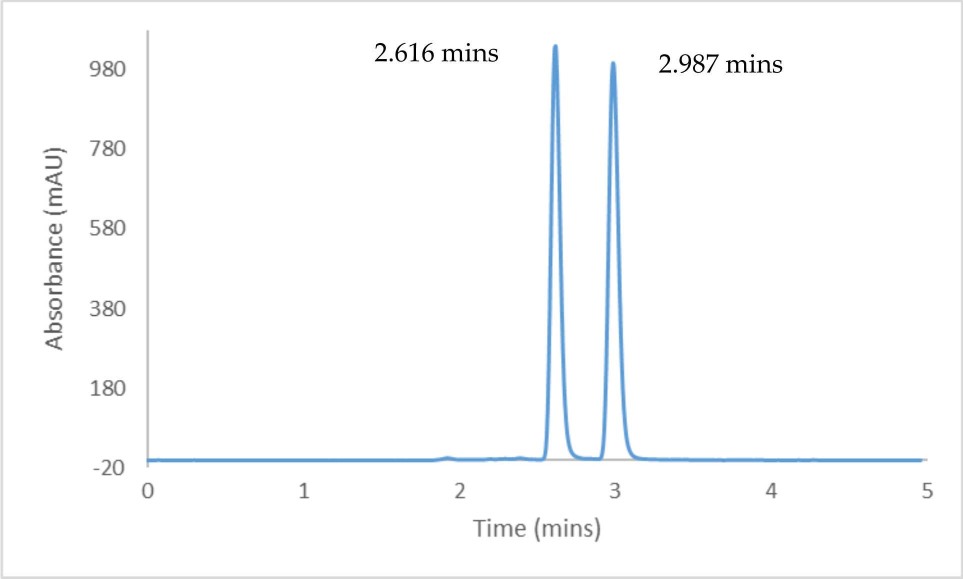

Figure S6: Separation of CBC on CHIRALPAK® IK-3 with Hex-EtOAc = 90-10 (v/v)

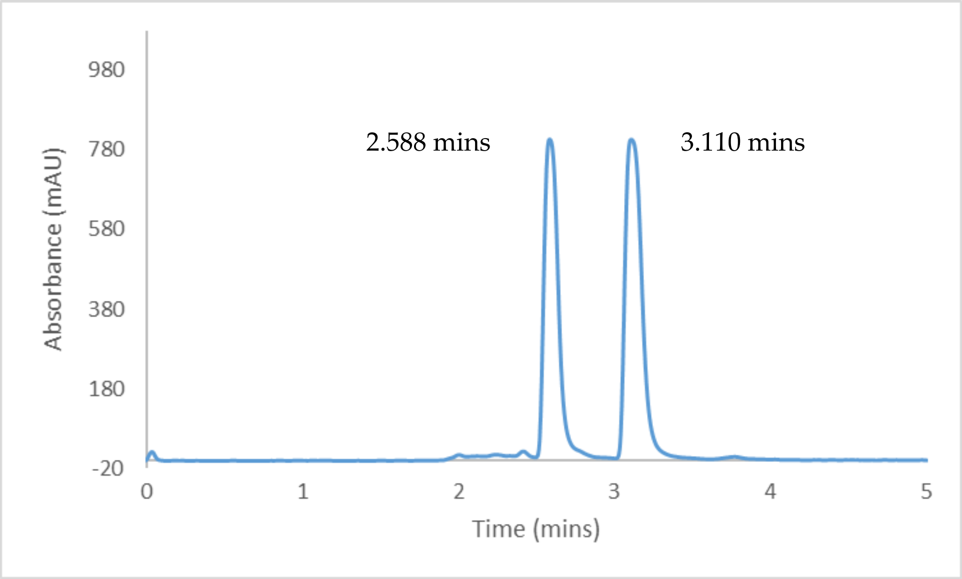

Figure S7: Separation of CBC on CHIRALPAK® IK-3 with Hex-MtBE = 80-20 (v/v)

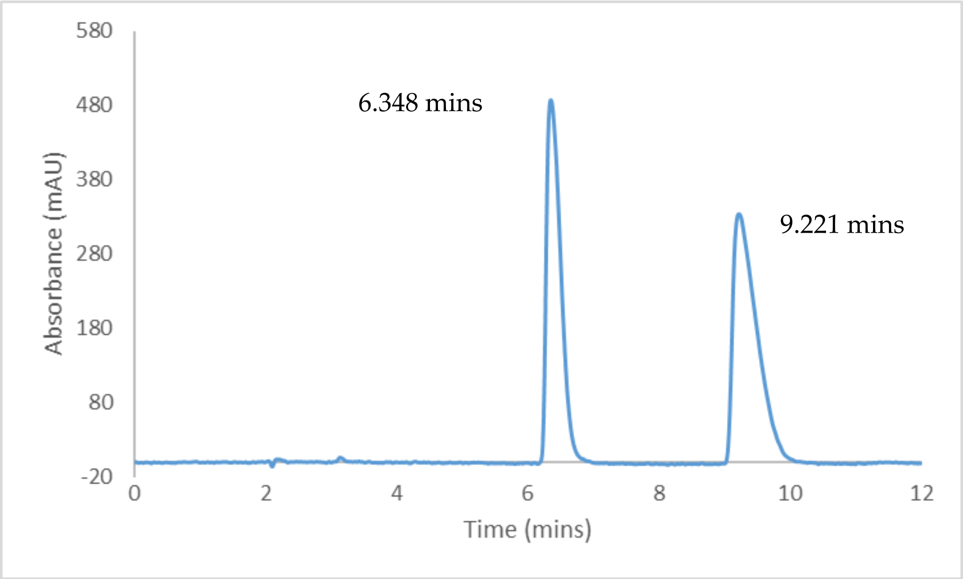

Figure S8: Separation of CBC on CHIRALPAK® IC-3 with Hex-DCM = 80-20 (v/v)

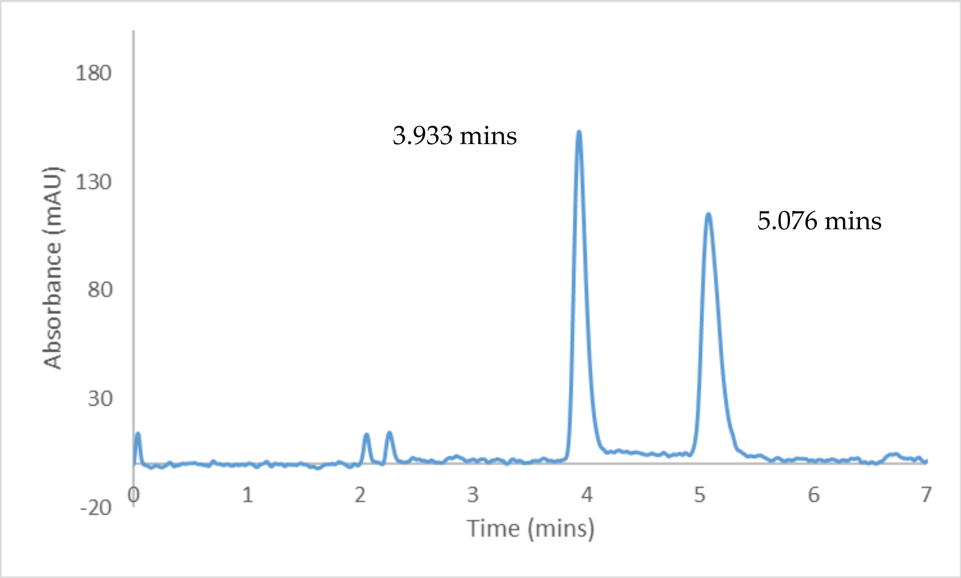

Figure S9: Separation of CBCA on CHIRALPAK® IC-3 with Hex-DCM-TFA = 80-20-0.1 (v/v/v)

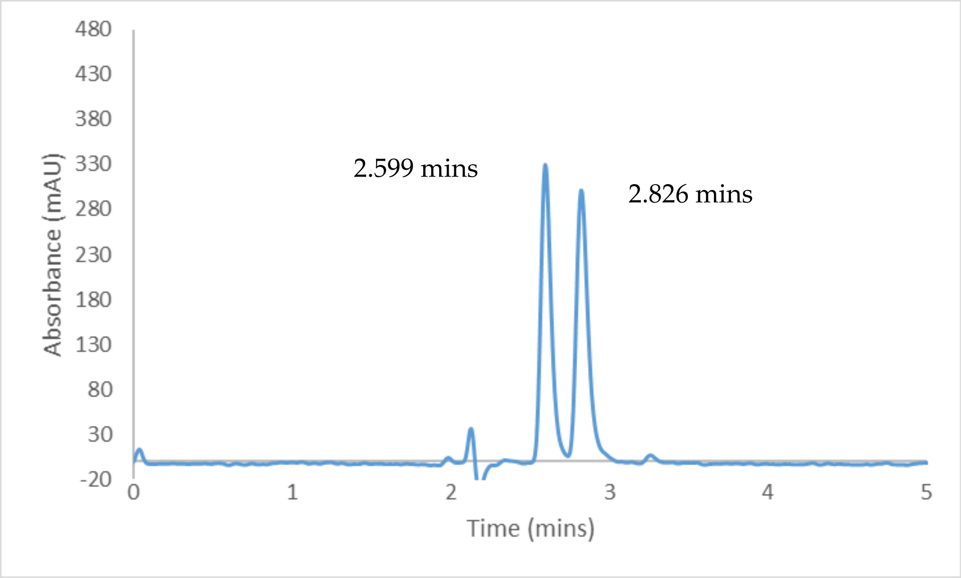

Figure S10: Separation of CBCA on CHIRALPAK® IK-3 with Hex-MtBE-TFA = 80-20-0.1 (v/v/v)

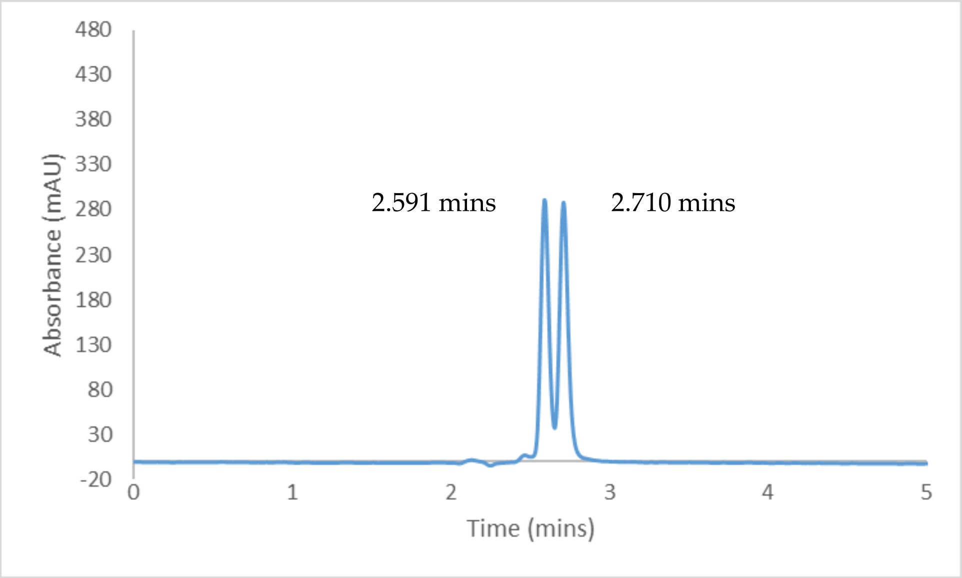

Figure S11: Separation of CBCA on CHIRALPAK® IK-3 with Hex-EtOAc-TFA = 90-10-0.1 (v/v/v)

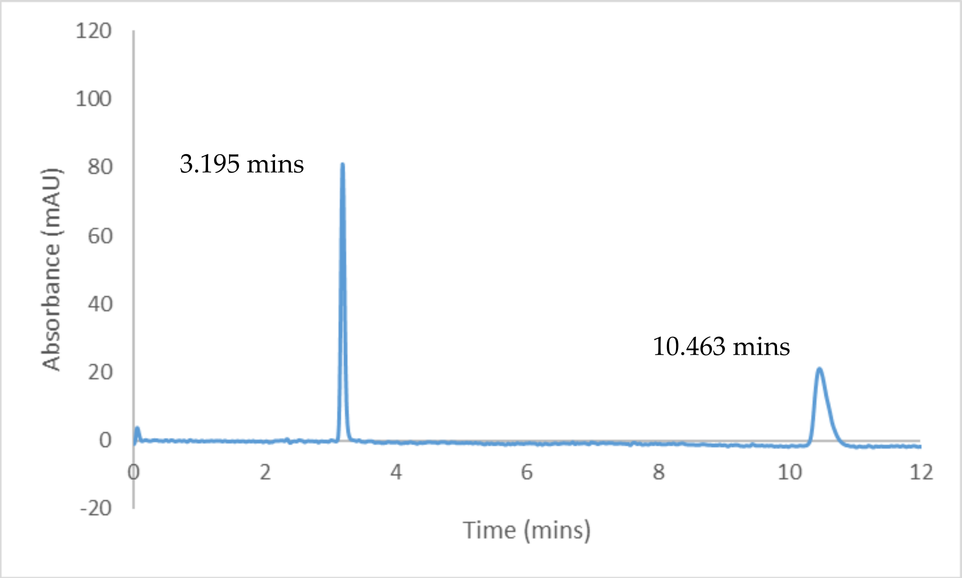

Figure S12: Separation of CBL on CHIRALPAK® IBN-3 with Hex-EtOAc = 90-10 (v/v)

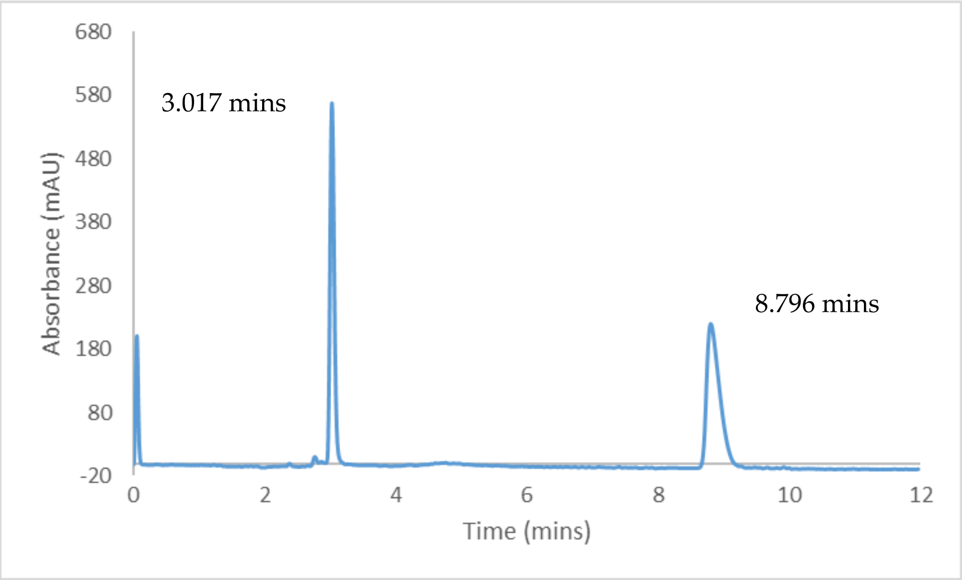

Figure S13: Separation of CBL on CHIRALPAK® IBN-3 with Hex-MtBE = 80-20 (v/v)

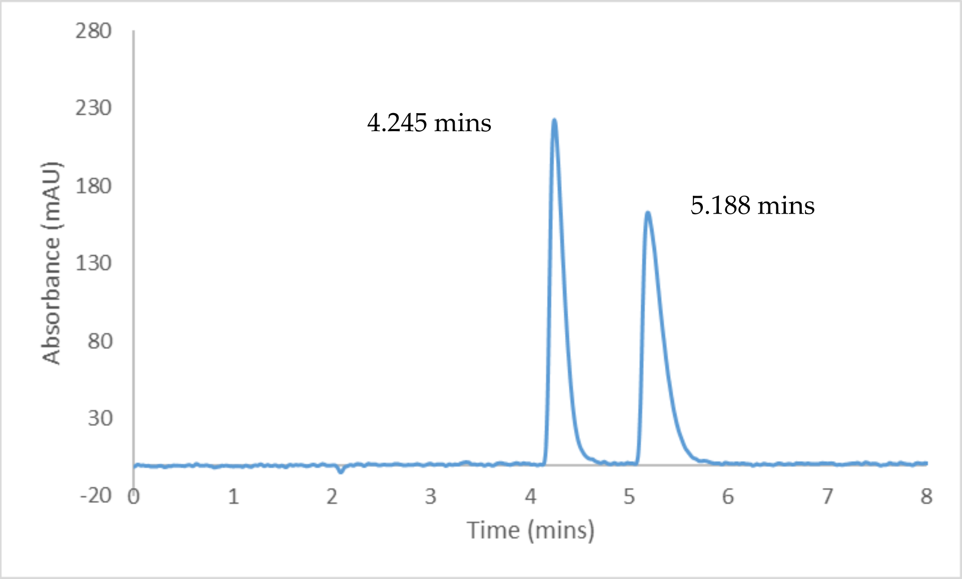

Figure S14: Separation of CBL on CHIRALPAK® IC-3 with Hex-DCM = 80-20 (v/v)

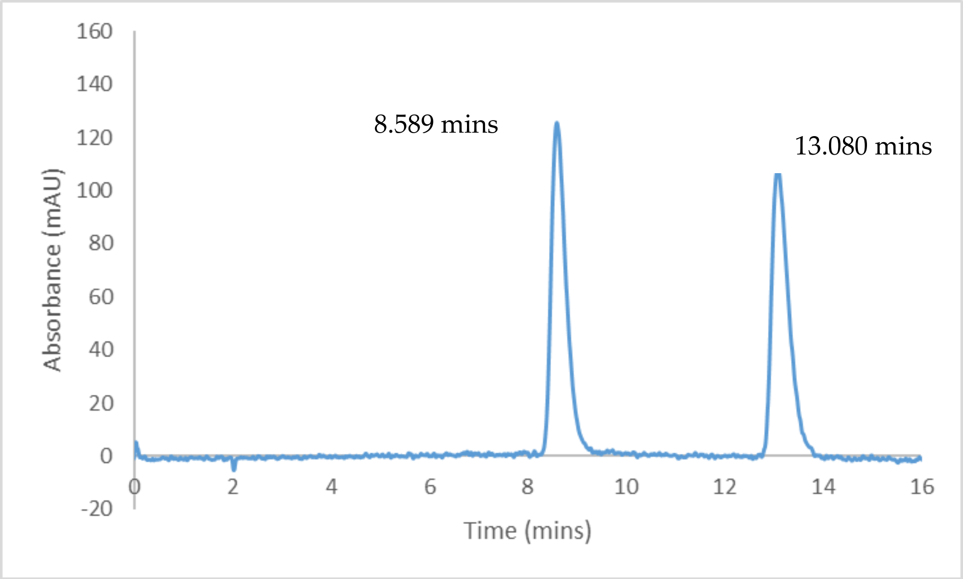

Figure S15: Separation of CBL on CHIRALPAK® IG-3 with Hex-DCM = 80-20 (v/v)

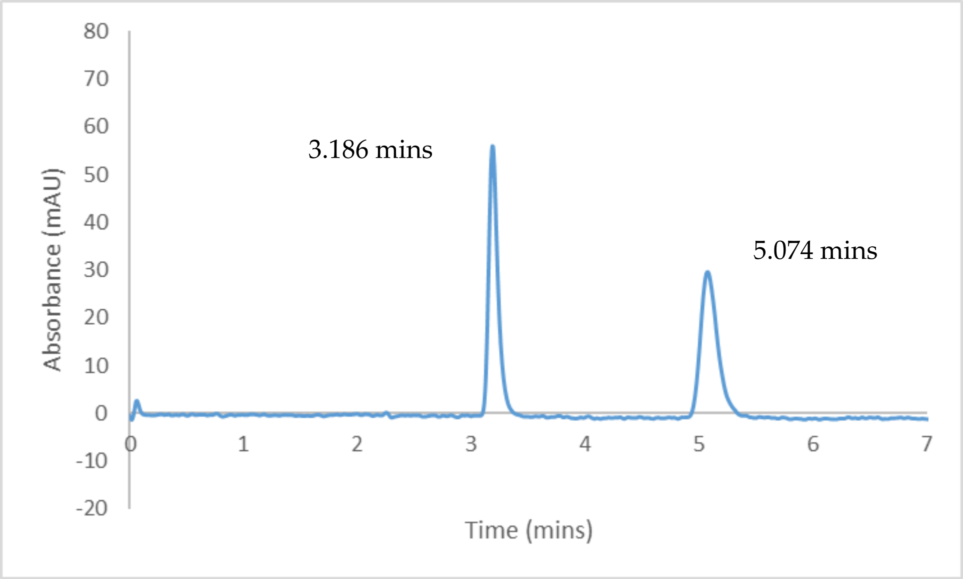

Figure S16: Separation of CBL on CHIRALPAK® IG-3 with Hex-EtOAc = 90-10 (v/v)

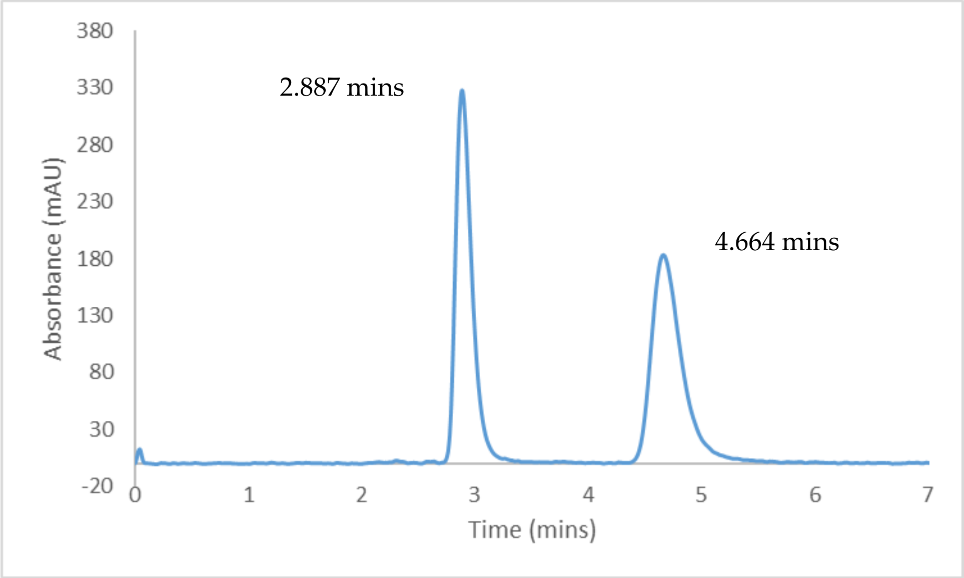

Figure S17: Separation of CBL on CHIRALPAK® IG-3 with Hex-MtBE = 80-20 (v/v)

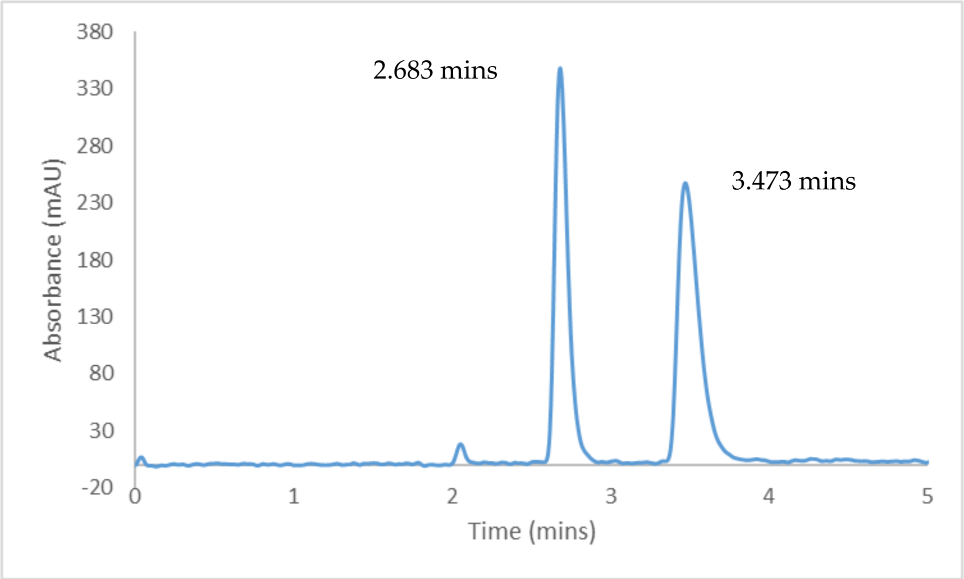

Figure S18: Separation of CBLA on CHIRALPAK® IC-3 with Hex-DCM-TFA = 80-20-0.1 (v/v/v)

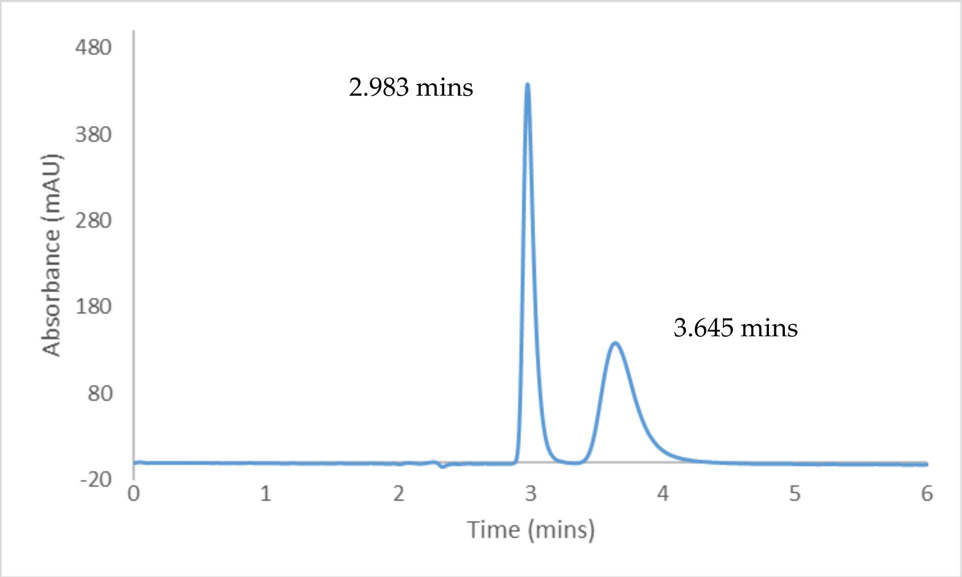

Figure S19: Separation of CBLA on CHIRALPAK® IG-3 with Hex-EtOAc-TFA = 90-10-0.1 (v/v/v)

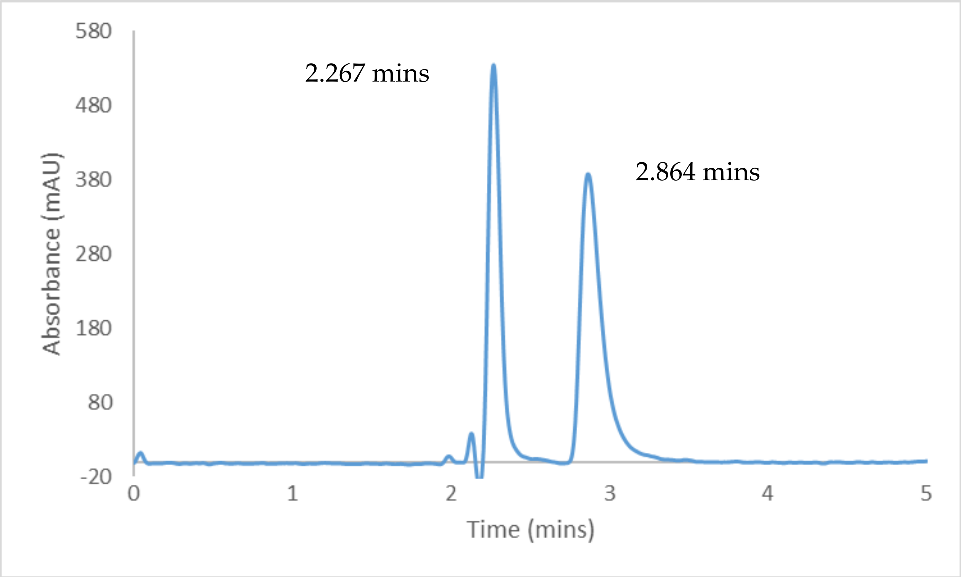

Figure S20: Separation of CBLA on CHIRALPAK® IK-3 with Hex-MtBE-TFA = 80-20-0.1 (v/v/v)
